# Deeper Profiles and Cascaded Recurrent and Convolutional Neural Networks for state-of-the-art Protein Secondary Structure Prediction

**DOI:** 10.1038/s41598-019-48786-x

**Published:** 2019-08-26

**Authors:** Mirko Torrisi, Manaz Kaleel, Gianluca Pollastri

**Affiliations:** School of Computer Science, University College Dublin, Belfield, Dublin 4 Ireland

**Keywords:** Protein sequence analyses, Protein structure predictions, Proteome informatics

## Abstract

Protein Secondary Structure prediction has been a central topic of research in Bioinformatics for decades. In spite of this, even the most sophisticated ab initio SS predictors are not able to reach the theoretical limit of three-state prediction accuracy (88–90%), while only a few predict more than the 3 traditional Helix, Strand and Coil classes. In this study we present tests on different models trained both on single sequence and evolutionary profile-based inputs and develop a new state-of-the-art system with Porter 5. Porter 5 is composed of ensembles of cascaded Bidirectional Recurrent Neural Networks and Convolutional Neural Networks, incorporates new input encoding techniques and is trained on a large set of protein structures. Porter 5 achieves 84% accuracy (81% SOV) when tested on 3 classes and 73% accuracy (70% SOV) on 8 classes on a large independent set. In our tests Porter 5 is 2% more accurate than its previous version and outperforms or matches the most recent predictors of secondary structure we tested. When Porter 5 is retrained on SCOPe based sets that eliminate homology between training/testing samples we obtain similar results. Porter is available as a web server and standalone program at http://distilldeep.ucd.ie/porter/ alongside all the datasets and alignments.

## Introduction

From DNA repair to enzyme catalysis, proteins are the chief actors within the cell. While we discovered more than 325 million protein sequences^1^, we still lack a feasible method to experimentally fully characterize them at a large scale. Nonetheless, nearly 150,000 protein structures are now freely available, growing by roughly 10,000 per year, making proteins a central research topic in Bioinformatics^2^. One of the most enduring open problems in Bioinformatics is Secondary Structure (SS) prediction^3,4^. It was inaugurated by Pauling and Corey in 1951, when they predicted the existence of the two most common SS conformations – *α*-helix and *β*-sheet – before the first protein structure was fully determined^5^. What followed was the first generation of SS predictors, all based on exploiting statistical propensities of single AA towards specific SS conformations^6–9^. The second generation of SS predictors was developed relying on information contained within segments of multiple adjacent amino acids (AA)^10^, physicochemical properties^11^, and algorithmic developments such as Neural Networks (NN)^12–14^, graph theory^15^, and nearest-neighbor methods^16^. Finally, the encoding of richer input including evolutionary information characterises the third generation of SS predictors, where profile-based inputs extracted from alignments of multiple homologous sequences led to accuracies exceeding 70% for the first time^17^. Notably, each generation of SS predictors has taken great advantage of the constantly growing availability of computational resources and data to exploit deeper information through more advanced methods^3,18^. Moreover, since the 90 s, NN have become the de facto standard technique to predict SS^12–14,19–26^, and maintain a central role at the two most important academic assessments of protein structure predictors: CASP and CAMEO^27,28^.

Six decades of efforts towards more accurate protein SS predictions have passed^4,29^. Nonetheless, the theoretical limits of prediction – set at 88–90% accuracy per AA, mainly due to the intrinsic dynamic nature of protein structure^30^ and ambiguity of SS class assignment – have not been reached yet, and the importance of accurate SS prediction as an intermediate step towards more complex protein features, such as tertiary, or quaternary structure, has not diminished^4,31^. We start this study assessing the potential and limits of SS prediction without evolutionary information, reaching roughly 70% accuracy, similar to that of early profile-based methods^17^. We then assess different NN architectures, focusing on classic window-based Feed Forward NN (FFNN) and cascaded Bidirectional Recurrent Neural Networks and Convolutional Neural Networks (CBRCNN) to gauge the relative strengths of each architecture. We investigate different pipelines to harness evolutionary information extracted with two of the most common tools – PSI-BLAST^32^ and HHblits^33^ – and benchmark different techniques to encode evolutionary information in the form of profiles. We develop a novel input encoding which is able to represent both evolutionary information and the identity of the query sequence. Finally, we implement the best methods into Porter 5, a state-of-the-art three- and eight-state SS predictor. Porter 5 is available as a light standalone program and a simple web server, alongside training and test datasets used for this study.

## Results

We trained profile-less and profile-based models with profiles encoded in a number of different ways. We identified the most successful predictors in 5-fold cross validation experiments on the training set. We ensembled some of these models in our final predictor Porter 5, which we tested on multiple independent sets alongside a number of the most recent SS predictors.

### Alignment-free predictions

Evolutionary information, in the form of aligned sequences, was first used to significantly improve the prediction of SS in the early 90 s^17^. The training sets used at the time contained only a few hundred proteins. For this study we were able to build a training dataset of almost 16,000 proteins (4 million AA). Given this massive growth in sample size, we tried to gauge whether it is now possible to produce reliable predictions without the use of alignments.

In 1993, an ensemble of 2 cascaded FFNN was adopted to reach a Q3 accuracy above 70% (see Methods: Measuring performances)^17^. We assessed window-based FFNN adopting an incremental training approach (described in Methods:FFNN) that allowed us to reach 69.7% Q3 accuracy with no profiles and just one hidden layer. We slightly improved the same FFNN up to 69.8% and 69.9% Q3 accuracy adding 1 or 2 hidden layers, respectively, our best results with a single FFNN without evolutionary information. It should be noted that a baseline predictor that classifies each residue into the most frequent secondary structure for its type results in a Q3 of 45.2%, 5.4% better than classifying all AA as the most common class (coils), see Table 1.

**Table 1:** Performances of single models of different NN architectures on the validation set.

Method	profile-less	plain profiles	deep profiles
Baseline	45.19%	60.9%	61.33%
FFNN	69.85%	80.02%	80.45%
CBRCNN	71.33%	82.32%	83.1%

To summarize, adopting a considerably larger training set but without evolutionary information, we reached comparable results to the 1993 state-of-the-art^17^. Using CBRCNN (see Methods:CBRCNN) instead of FFNN we observed a further increase in accuracy, up to 71.3% on the same sets. While there might be advantages to alignment-less predictions, as they require considerably less computational time with respect to profile-based solutions (fractions of seconds per protein instead of minutes), their accuracy, at ~71%, is far from the state-of-the-art predictors including evolutionary information, estimated at ~82–83%^29,34^.

### Profile-based predictions

In a second phase of this study we tested different ways to encode alignments in order to maximise input information to a predictor.

We generated alignments with both PSI-BLAST^32^ and HHblits^33^. We did not limit the number of hits of PSI-BLAST or HHblits, resulting in alignments with an average of ~14,000 and ~1,300 proteins, respectively (see also Methods:Evolutionary Information). We encoded evolutionary information into 22 inputs using plain profiles, as described in Methods:Input Encoding.

We trained a three hidden layer FFNN constructed similarly to the best FFNN based on single-sequence inputs. We obtained 79.9% accuracy, a 10% improvement over the alignment-less case. Fine-tuning the FFNN hyperparameters did not substantially change the results, with only slight improvements for networks with larger hidden layers, confirming that there is more information embedded in a profile than in a single sequence.

It should also be noted that, when using profiles, we obtain a Q3 accuracy of 60.9% completely disregarding the context surrounding an AA (training a FFNN with window of size 1), see Table 1.

CBRCNN performed significantly better on the same data and encoding, up to 82.3% for a single model, matching the performance of a fully tuned ensemble trained on a smaller set^34^.

#### Deeper profiles

We found beneficial to employ, at encoding time, a weighting scheme that aims to maximize the entropy of the profiles (see Methods:Input Encoding), i.e. that weighs more those sequences that are more informative (more different from the plain profile). This step improved the Q3 accuracy of our best FFNN and CBRCNN by 0.4% and 0.3%, respectively, while maintaining an encoding composed of 22 input numbers per AA.

We trained both FFNN and CBRCNN on several more encoding schemes (not reported), testing various options of PSI-BLAST and concatenating additional features such as protein length or the encoded sequence without a profile from alignments. We obtained the best results by adopting a simple, novel “clipping” technique (see Methods:Input Encoding) that is capable of presenting both the weighted profile from the aligned sequences and the identity of the AA in the protein itself, while keeping the encoding size unchanged. Combining this clipping scheme and the alignment profile of maximal entropy, a single CBRCNN reached 83.1% Q3 accuracy, as reported in Table 1.

#### HHblits

As a final step to exploit evolutionary information, we adopted alignments generated by HHblits^33^, and compared it to PSI-BLAST^32^.

Although HHblits aligns considerably fewer sequences in our experimental settings (roughly a tenth of PSI-BLAST, that is ~1,300 proteins in our case), the set of hyperparameters selected for the CBRCNN trained on PSI-BLAST also worked close to optimally for training on HHblits inputs. In particular, after some tuning of the HHblits options (see Methods:Evolutionary Information), we observed a Q3 accuracy of 83.15% training CBRCNN on HHblits inputs, directly comparable with the 83.1% obtained on PSI-BLAST inputs. Refining on HHblits profiles models previously trained on PSI-BLAST gave a Q3 accuracy of 83.41%. Training a single CBRCNN on the average of PSI-BLAST and HHblits inputs improved the accuracy further, to 83.79%. We found less beneficial to train on inputs encoded from the union (83.41%) or the intersection (82.81%) of the two sets of alignments. Finally, we obtained 83.77% Q3 accuracy training on the concatenation of PSI-BLAST and HHblits profiles (44 inputs rather than 22). See Table 2 for a summary.

**Table 2:** Performances of single CBRCNN trained with different approaches relaying on both PSI-BLAST and HHblits.

Training	*From scratch*	Refining	Average	Union	Intersection	Concatenation
Q3 Accuracy	*83.15*%	83.41%	83.79%	83.41%	82.81%	83.77%

### Towards state-of-the-art predictor

Finally, we built an ensemble of predictors based on the most successful individual models. All the experiments were run on five-fold cross-validation to gauge generalization performances of the ensemble^35^.

#### Ensembling

Bayesian model averaging is a classic ensembling approach which we exploited since the first version of Porter^22^: the outputs of individual models (outputs of a softmax function in our case) are simply averaged component by component. We ran preliminary testing by splitting our set into 1/5 for testing and 4/5 for training. An ensemble of the best 17 CBRCNN, with different hyperparameters but all trained on PSI-BLAST, achieved an accuracy of 84% (Table 2). Ensembles of decreasing sizes record modest reductions in performances, down to 83.82% with just 3 CBRCNN. Adding 3 structurally identical CBRCNN trained on HHblits inputs we observed a Q3 accuracy of 84.63%. We could not significantly improve on this by adding any further model trained on either HHblits or PSI-BLAST. Adding to the ensemble the single best performing CBRCNN trained on the concatenation of PSI-BLAST and HHblits inputs led to a further small increase in performances, up to 84.7% Q3.

We then tested this same ensemble of 7 models in 5-fold cross-validation, without any further tuning of hyperparameters or any change in the models selected. We obtained very similar results to our preliminary testing. The overall ensemble accuracy, averaged over the 5 folds, was 84.85%.

Finally, we trained from scratch the 7 best performing CBRCNN (selected by cross-validation, as described above) on the full training set rather than on individual training folds of the cross-validation. We then tested an ensemble of these 7 models on a completely independent set (see “2017_test” in Methods:Datasets) containing over 3,000 proteins. We compared the accuracy of this ensemble against the ensemble of all 35 models resulting from the 5-fold cross-validation training (3 PSI-BLAST CBRCNN, 3 HHblits CBRCNN and 1 PSI-BLAST + HHblits CBRCNN for each of the 5 folds). As reported in Table 3, while there were some differences between the accuracies of individual components of these two solutions, the overall ensembles performed almost identically, hence the retrained ensemble of 7 models is preferable for the final predictor as it is computationally more compact than the ensemble of 35 models.

**Table 3:** Assessment on the 2017_test set of three-state ensembles trained on either five-fold cross-validation or full set.

Training strategy	PSI-BLAST	HHblits	Concatenation	PSI-BLAST and HHblits	All the previous
Five-fold cross-validation	83.55%	83.55%	83.98%	84.18%	84.19%
Full set (Porter 5)	83.42%	83.39%	83.49%	84.13%	84.19%

#### Stacking and further results

We tried many other architectural solutions during preliminary testing, including deep FFNN architectures, and structures akin to Residual Neural Networks^36^, in which the global inputs to the model (the profile of residue frequencies) is presented to downstream stages through shortcut connections alongside the predictions of previous stages^37^. While we observed small improvements when modestly increasing the number of hidden layers (up to 3–4, depending on the precise configuration), the results we obtained were generally poorer than those we observed with CBRCNN - typically around 1.5% worse than individual CBRCNN of similar size, and approximately 2% worse than those of a stack of 2 CBRCNN, which is what we used in our final predictors. While it is not entirely clear why, it appears that the recurrent stages in the CBRCNN are more efficient at capturing the sequential dynamics of our inputs than those of feed-forward networks alone, possibly because of their unrestricted input size. We did observe only marginal improvements in performances (roughly +0.1%) when stacking more than 2 CBRCNN stages with shortcut connections, and decided against including these more complex models into our final testing and predictor.

### Eight-state prediction

We applied the same pipeline described in Ensembling (section above) to the prediction of the full DSSP 8-class definition of SS^38^. It should be noted that this slightly increases the total number of tunable parameters of the CBRCNN with respect to three-state SS prediction. In particular, we applied Bayesian model averaging on an equal number of CBRCNN trained on either PSI-BLAST or HHblits inputs, and some trained on concatenated inputs (as in Table 2). We obtained 71.76%, 71.66% and 72.29% Q8 accuracy training single CBRCNN on 4/5 of the training set on PSI-BLAST, HHblits and concatenated inputs, respectively. An ensemble of 3 CBRCNN trained on PSI-BLAST inputs yields 72.47% Q8 accuracy on the same fold. When we add to the input of these networks the output of the ensemble of the 3 corresponding (PSI-BLAST) models trained on the 3-class problem we record a further improvement, to 72.79% Q8 (+0.3%), without dramatically increasing the encoding size (total of 25 inputs). We extended this approach to the 3 HHblits-trained models and to the one trained on concatenated PSI-BLAST and HHblits profiles.

The overall ensemble of 7 models trained on the full training set achieves 73.02% Q8 accuracy on the 2017_test set described in Methods:Dataset (see also Table 4). An ensemble of the same 7 CBRCNN without the three-state predictions as inputs has an accuracy of 72.11%, confirming that including these predictions is beneficial.

**Table 4:** Q3/Q8 accuracy and SOV score per AA on the full test set.

Method	Q3	SOV’99	SOV_refine	Q8	SOV8’99	SOV8_refine
**Porter 5**	**84.19%**	**81.19%**	**76.72%**	**73.02%**	**69.91%**	**72.09%**
Porter 5 (HHblits and PSI-BLAST)	83.49%	80.17%	75.64%	71.94%	69.03%	71.45%
Porter 5 (PSI-BLAST only)	83.42%	80.41%	75.8%	72.11%	69.28%	71.56%
Porter 5 (HHblits only)	83.39%	80.19%	75.59%	71.8%	68.87%	71.16%
SSpro 5.1 with templates	82.62%	79%	74.58%	71.91%	68.68%	70.72%
PSIPRED 4.01	82.06%	77.83%	72.95%	N.A.	N.A.	N.A.
RaptorX-Property	82.04%	78.57%	73.66%	70.74%	67.59%	69.65%
Porter 4	82%	78.85%	73.89%	N.A.	N.A.	N.A.
DeepCNF	81%	76.96%	71.84%	69.76%	66.42%	68.5%
SSpro 5.1 ab initio	80.7%	76.85%	72%	68.85%	65.33%	67.54%

### Assessment of multiple predictors on independent test set

Porter 5 is an ensemble of 7 CBRCNN (see Ensembling, above): 3 trained on PSI-BLAST, 3 trained on HHblits and 1 trained on both (44 inputs rather than 22). Porter 5 relies on 7 more CBRCNN to predict eight-state SS (see Eight-state prediction, above). We tested Porter 5 against Porter 4^34^, Spider3^26^, SSpro 5.1^24^, PSIPRED 4.01^20^, RaptorX-Property^39^ and DeepCNF^25^ on the 2017_test set we created, containing 3,154 proteins. Spider3 rejects proteins containing undetermined (X) amino acids (562 overall) and, when we use the parameters required by Spider3, either PSI-BLAST or HHblits do not return a valid result for 129 proteins. Because of this we report results on two sets: one where we exclude the proteins on which we could not obtain a valid response from Spider3 (2,463 entries composed by 497,142AA) (Table 5); the full set of 3,154 proteins (Table 4) on which Spider3 is not assessed.

**Table 5:** Performances on the smaller 2017_test set for which Spider3 generates predictions, sorted by Q3 accuracy.

Method	Q3 per AA	SOV’99 per AA	SOV_refine per AA	Q3 per protein	SOV’99 per protein	SOV_refine per protein
**Porter 5**	**83.81%**	**80.41%**	**75.73%**	**84.32%**	**81.05%**	**76.45%**
Spider3	83.15%	79.43%	74.68%	83.42%	79.79%	75.07%
Porter 5 (HHblits only)	83.06%	79.49%	74.71%	83.68%	80.26%	75.58%
SSpro 5.1 with templates	82.58%	78.54%	74.02%	83.94%	80.29%	76.15%
PSIPRED 4.01	81.88%	77.36%	72.33%	82.48%	78.22%	73.31%
RaptorX-Property	81.86%	78.08%	72.99%	82.57%	78.99%	74.03%
Porter 4	81.66%	78.05%	72.89%	82.29%	78.61%	73.55%
SSpro 5.1 ab initio	81.17%	76.87%	72.03%	81.1%	76.92%	72.12%
DeepCNF	81.04%	76.74%	71.47%	81.16%	76.99%	71.7%

Porter 5 is the most accurate 3-state and 8-state predictor in our tests on the 2017_test set with 3-class accuracy of 83.8% on the smaller version of the set and 84.2% on the larger one, 0.7% better than Spider3, 1.2–1.6% better than SSpro 5.1 with templates, and at least 2% more accurate than all the other predictors.

Performances of the servers show very similar deviations and differences greater than approximately 0.12% in Table 4 and 0.14% in Table 5 are significant at p = 0.05. Porter 5 is also very fast given the small size of its models (on average 39k parameters for the 3-class networks, 58k for 8 classes). Once the alignments by PSI-BLAST and HHblits are present, Porter 5 runs 2 orders of magnitude faster than Spider3.

We also measured the SOV’99^40^ and the SOV_refine^41^ (see Methods:Measuring performances) of every SS predictor on both versions of the 2017_test set. Porter 5 is consistently the best-performing 3-state and 8-state SS predictor, with both SOV scores at least 1% and 2% better than any other SS predictor on small and large versions of the set, respectively. Porter 5 is also 1.2% better that any other predictor considering the 8-state SOV’99 and the SOV_refine scores.

Finally, we measured Porter 5 performances on the CASP13^28^ set, and on 6 months of proteins (December 28 2018 to June 22 2019) released by CAMEO^27^. The results are reported in Table 6 and roughly confirm the Porter 5 results we obtained on the 2017_test set.

**Table 6:** Assessment of Porter 5 on CASP13, i.e. 43 targets, and on the last 6 months of CAMEO, i.e. 463 proteins released from Dec 28, 2018 to Jun 22, 2019.

Method	Q3	SOV’99	SOV_refine	Q8	SOV8’99	SOV8_refine
CAMEO	85.48%	82.08%	78.08%	74.99%	72.36%	74.81%
CASP13	82.99%	78.36%	73.39%	71.08%	66.95%	69.27%

#### Nuclear magnetic resonance

We also analyzed the performance of Porter 5 separately on proteins resolved by Nuclear Magnetic Resonance (NMR) and by X-ray crystallography. NMR proteins are predicted at a significantly lower Q3 accuracy (81.6%, *σ* = 0.12%), possibly because of their different statistics (e.g. average length and composition) or less certain determination of SS. The X-ray only section of the 2017_test set, which is roughly 90% of the total, is predicted at an average Q3 of 84.65% (Table 7).

**Table 7:** Porter 5 on NMR vs X-ray crystallography proteins.

Method	Q3	SOV’99	SOV_refine	Q8	SOV’99	SOV_refine
NMR	81.61%	76.64%	70.55%	67.52%	62.41%	63.86%
**X-ray**	**84.65%**	**81.99%**	**77.81%**	**74%**	**71.24%**	**73.54%**

#### Porter 5 with SCOP based redundancy reduction protocol

While redundancy reduction protocols similar to the one we adopted to build our sets are widely used^17,21,22,26,34,39,42–44^, this type of redundancy reduction does not fully eliminate the occurrence of proteins with similar 3D structures (hence similar SS) in the training and testing sets^20,45^. Normally, the only way to genuinely control for this and produce sets that are completely devoid of structurally homologous examples is to resort to classifications of protein structures such as SCOPe/ASTRAL^46^ and use information gleaned from these to guide the construction of the data sets, e.g. by selecting only one representative per superfamily or family of proteins. The drawback of this procedure is that the resulting sets will be smaller, and it has been shown in different occasions (e.g.^34^) that, all other factors being the same (e.g. same algorithms, same redundancy reduction protocols) larger data sets lead to improvements in performances. In order to gauge the effect of stricter redundancy reduction criteria on the methods presented here, we retrained 2 separate versions of Porter 5 using the JPred4 sets^45^. In these sets, only one representative for each of the 1,358 SCOPe/ASTRAL v.2.04^46^ superfamily domain sequences is selected for the training set, while a further 150 proteins from superfamilies not included in the training set are used as a blind test set. The first version we retrained uses the exact same protocol and ensemble as Porter 5, including recent versions of the UniRef database for the creation of MSA and a combination of alignments by PSI-BLAST and HHblits, but it is trained on the 1,348 JPred4 set. In the second version we also adopted the same alignments used by JPred4, based on release 2014_7 of UniRef90 and obtained using PSI-BLAST, which are available from the JPred4 web site. It should be noted that in this case, given that we do not use HHblits alignments, all the models in the ensemble are trained solely on PSI-BLAST profiles. The first version, adopting recent alignments, achieves 84.62% correct prediction on the JPred4 blind set. The second version, which relies on the exact same training and testing data as JPred4, achieves 83.62% correct prediction. JPred4, which is based on a standard feed-forward neural network architecture, has a 82.29% Q3 per amino acid on the same sets. While the testing set is small (150 proteins), these results suggest that more sophisticated machine learning algorithms (and, indeed, more up to date alignment sets and treatment thereof) may be beneficial to predictive performances. These results also roughly match what we found on our larger data sets, although it should be noted that the class definition in these sets is slightly different in that DSSP class ‘G’ is assigned to Coil rather than to Helix. This different assignment has been shown in the past to lead to somewhat higher Q3 values, e.g. in^47^, which might explain how Porter achieves a similar Q3 when trained on a set which is an order of magnitude smaller than its original training set.

We also assessed Jpred4 on the 2019_test set (see Table 8 and description of the set in the following section) with classes recast to match the Jpred4 class assignment. In this case the more modern predictors including Porter 5 show Q3 3.7–4.7% higher than Jpred4 and similar improvements in SOV, suggesting that larger training sets, alongside larger alignment sets and more sophisticated algorithms, may be beneficial.

**Table 8:** Most recent predictors and Jpred4 assessed on 2019_test set of 618 proteins.

Method	Q3	SOV’99	SOV_refine	Q8	SOV8’99	SOV8_refine
SPOT-1D	82.13%	76.65%	71.37%	69.69%	65.52%	67.18%
**Porter 5**	**81.74%**	**76.67%**	**71.03%**	**68.25%**	**63.24%**	**64.87%**
NetSurfP-2.0	81.3%	75.64%	70.3%	67.93%	62.77%	64.66%
MUFOLD-SS	81.09%	75.28%	69.87%	68.21%	64.3%	66.33%
*Jpred4*	*77.38%*	*72.29%*	*64.96%*	*N.A*.	*N.A*.	*N.A*.

### Assessment of latest SS predictors

In a separate test set we assessed some very recent predictors which have been trained on sets more recent than our 2017_test set, i.e. MUFOLD-SS^43^, NetSurfP-2.0^48^ and SPOT-1D^44^. Because of this, we generated a second independent test set (also see Methods:Datasets) starting from June 24 2019 PDB proteins, 25% redundancy reduced against the Porter 5, NetSuft-2.0 and SPOT-1D training sets (we could not access the MUFOLD-SS training set). As either MUFOLD-SS or SPOT-1D or both did not produce a valid prediction for 243 out of the 861 original proteins in the set, Table 8 shows the performances observed on the 618 proteins successfully predicted by all predictors of this group. The 3 predictors have similar performances, with Porter 5 slightly outperforming both MUFOLD-SS and NetSurfP-2.0, but being slightly outperformed by SPOT-1D. Differences of 0.28% in Q3 and 0.37% in Q8 in the table are significant at p = 0.05. It should be noted that SPOT-1D relies on the predictions of SPOT-Contact^49^, i.e. a Contact Map predictor^4^, which in turn requires Spider3^26^, CCMpred^50^, and DCA^51^. This results in a far more computationally intense pipeline which in essence derives the SS from a guess of a protein’s 3D structure through its contact map^4^.

## Discussion

In this study we describe the development of a new, state-of-the-art SS predictor, Porter 5^52^. We trained window-based feed-forward neural networks with different hyperparameters and input encoding (see Table 1 to define our baselines and assess the quality of our large training set (see Table 9). We developed both a state-of-the-art model, and a novel encoding technique, i.e. “clipping”. We assembled the final predictor Porter 5 as a simple ensemble of models trained on different inputs, i.e. either PSI-BLAST^32^ or HHblits^33^ or a concatenation of both. We applied a very similar approach to the harder eight-state SS prediction problem to develop the eight-state version of Porter 5 which represents, analogously to the three-state Porter 5, the state-of-the-art for this task (see Table 4). Porter 4, the previous release of Porter, was trained on 7,522 proteins^34^. Thanks to the constant growth of the PDB^2^, we performed all the experiments on a training set twice as large as the one adopted for Porter 4, i.e. 15,753 proteins (see Methods:Datasets). The results we present in this study confirm the continuing positive contribution of a larger, well-distributed training set.

**Table 9:** Overview of AA composition of Training, 2017_test and 2019_test.

3-states	8-states	Training Set		2017_test Set		2019_test Set	
Helices	G	38%	135,498	39.22%	21,404	37.41%	2,300
	H		1,306,610		233,961		31,854
	I		714		177		33
Sheets	E	22.15%	800,297	20.9%	129,425	17.87%	15,411
	B		41,026		6,793		916
Coils	C	39.85%	764,391	39.88%	133,183	44.72%	21,605
	S		331,075		57,678		9,804
	T		417,815		68,973		9,452

For this study, we exploited evolutionary information through different encodings and gauged their importance with respect to an encoding containing only the plain protein sequence. While we can now predict SS using plain protein sequences at an accuracy that would have represented the state-of-the-art including evolutionary information 25 years ago, we observed that evolutionary information is as important as ever, boosting prediction accuracies by 10% or more. In particular we used evolutionary information mined by both PSI-BLAST^32^ and HHblits^33^ and observed that while they lead to broadly similar predictive accuracies when used individually, their combination is clearly beneficial. To the best of our knowledge, Porter 5 is the first SS predictor to ensemble models trained on PSI-BLAST or HHblits, which (empirically) appears to be the most effective way to exploit both algorithms at the same time (see Table 2).

We have also studied a number of different models, confirming that recurrent neural network architectures are particularly effective at SS prediction, with a combination of bidirectional recurrent networks and dense convolutional layers being the best performing model. While a modest increase in the number of stages adopted worked well for us, we did not observe improvements in performances beyond 3–4 internal layers for feed-forward networks and 2 stages of BRNN-CNN stacks. This seems to suggest that, at least given the current sizes of training sets, recurrent neural network stages capture all the long-range information that can be exploited effectively.

Unlike many other modern “deep” predictors, Porter 5’s models are individually tuned to have roughly correct individual expressive power rather than being oversized in the first place and kept to the right capacity by regularization techniques or dropout. Individual models within Porter 5 have 40,000–60,000 free parameters. This is significantly less than the average 500,000 parameters of DeepCNF^25^, i.e. the PSI-BLAST version of RaptorX-Property^39^, or the well over one-million of Spider3^26^, although it is still a 2–3 fold increase with respect to the 13,000–18,000 free parameters of Porter 4^34^. The relative small size of Porter 5 also means that, once alignments are available, individual predictions are extremely fast to run.

We assessed Porter 5 on a first independent test set (2017_test), along with some of the SS predictors trained up to 2017: DeepCNF^25^, Porter 4^34^, PSIPRED 4.01^20^, RaptorX-Property^39^, Spider3^26^ and both versions of SSpro 5.1^24^, i.e. profile-based and template-based. In all our tests Porter 5 outperformed the other methods, often by large margins with an accuracy of approximately 84% for 3-class SS prediction and 73% for 8-class prediction. It should also be noted that our assessment might be somewhat optimistic for some of the competing predictors since we did not perform any redundancy reduction of our final test set against their training sets^35^.

Finally, we assessed Porter 5 against some of the most recent predictors which have also been trained on very recent and large training sets, i.e. MUFOLD-SS^43^, NetSurfP-2.0^48^ and SPOT-1D^44^. As these predictors’ training sets overlapped with our original test set, we generated a second smaller independent test set (2019_test) based on PDB sequences uploaded up to June 24th. In this case we observed results which are broadly similar between these newer predictors, with Porter 5 slightly outperforming both MUFOLD-SS and NetSurfP-2.0, but slightly outperformed by SPOT-1D which, however, is built on a more complex and computationally intensive (though highly effective) pipeline in which the SS is predicted through a protein’s contact map.

## Methods

### Datasets

The selection and preparation of datasets to adopt has a central role in any machine learning method^35^. We built our datasets from the Protein Data Bank (PDB)^2^, the public repository of all the freely and publicly known protein structures. We assembled our final datasets only with proteins sharing up to 25% sequence identity^35^. Specifically, we built our training set from the PDB released on Dec 11 2014, internally redundancy-reduced at a 25% identity threshold. We also built an independent test set (2017_test) from the PDB released after Dec 11 2014 and up to Jun 14, 2017. We redundancy-reduced this set at a 25% identity threshold against the training set. Further, we internally redundancy reduced the resulting set at a 25% identity threshold. Finally, we removed all proteins with at least 10 consecutive undetermined AA from both sets. The training set contains 15,753 proteins (3,797,426 AA) and 2017_test 3,154 proteins (651,594 AA), among the largest ever used to build a SS predictor. The SS states were assigned according to the Dictionary of Protein SS (DSSP)^38^ and their distribution is highlighted in Table 9. In different tests the training set is used as a whole for training purposes or split into 5 randomly distributed folds in cross-validation for hyperparameter optimization^35^. The 2017_test set is only used in the final part of this study to evaluate our final solutions and other solutions previously published. The training and the test sets are available at http://distilldeep.ucd.ie/porter/.

We also curated an additional independent test set (2019_test) to fairly compare Porter 5 against some of the most recent SS predictors, i.e. MUFOLD-SS^43^, NetSurfP-2.0^48^ and SPOT-1D^44^, which have been trained on sets overlapping with our 2017_test set. We removed any protein shorter than 30 AA or containing more than 10% of undetermined AA from the PDB proteins deposited up to Jun 24 2019. We then redundancy-reduced this set against the training sets of SPOT-1D, NetSurfP-2.0 and our training set at 25% identity threshold. Finally, we reduced the internal redundancy of this set at a 25% sequence identity threshold and obtained 861 proteins. As MUFOLD-SS or SPOT-1D or both do not return a valid answer for 243 of these proteins, we report results on 618 proteins, comprising 91,375 amino acids (2019_test).

### Evolutionary information

A key aspect of any modern SS predictor is harnessing evolutionary information^53^. PSI-BLAST^32^ and more recently HHblits^33^ are methods widely used for the purpose – i.e. gathering known protein sequences which are likely to be evolutionarily related to the protein of interest^54^. We relied on both, finding the best results with the default settings and iterating them 3 times with an e-value of 0.001^55^ without limiting the number of sequence hits. PSI-BLAST is run on the May, 2016 version of UniRef90^1^, containing almost forty-two millions clusters. HHblits is run on the February, 2016 version of UniProt20, containing over eight millions clusters. Our experiments show similar results when a model is trained with either PSI-BLAST or HHblits, but significant improvements when both are used (see Results:HHblits).

#### Input encoding

Among the several encoding schemes assessed, we focused on three approaches: alignment-free, plain profiles and weighted profiles.

For the alignment-free case, when no evolutionary information is employed, we adopted a simple one-hot encoding of 20 positions - one for each standard AA - and a zero vector for non standard AA, i.e. “B”, “J”, “O”, “U”, “Z”, and “X”.

For the plain profiles case, our baseline for employing evolutionary information, we adopted arrays of 22 positions composed of 20 frequencies for standard AA, 1 for unknown or non-standard and the last position for gaps. The first 21 numbers are normalized to add up to 1 without considering gaps, while the 22nd number represents the total frequency of gaps in a column of the alignment.

For the weighted profiles case, we maximized the entropy deriving from the evolutionary information applying a weighting scheme to the plain profiles^56,57^. In particular, we calculated the weight of each sequence in the alignment as:

$${W}_{seq}=\mathop{\sum }\limits_{n=1}^{length}\,-\,\mathrm{log}\,f[a{a}_{seq}(n)]$$

where *f*[*aa*_*seq*_(*n*)] is the relative frequency of the n-th AA of sequence *seq* within column *n* of the alignment. We then weighted every sequence *seq* in the alignment by W_*seq*_ and, finally, normalized as for the plain profiles case, i.e. the first 21 components add up to 1 and the 22nd is normalized independently. Differently from plain profiles, we did not consider external gaps when calculating the gap frequency.

Clipping is the novel encoding method we introduce in this study. The simple idea is to set to 1 the position in the profile vector associated to the AA in the query sequence, regardless of its frequency in the alignment. This approach can be seen as a merging technique between one-hot encoding – adopted when evolutionary information is lacking – and any method to represent evolutionary information. It should also be noted that no information in the profile is lost when adopting clipping, as any one of the 21 numbers in the profile is equal to 1 minus the sum of the others.

We also build a version of Porter 5 which predicts the eight-state SS classes by the DSSP program^38^. In this case the output of the three-state Porter 5 is concatenated to the input – i.e. 25 inputs rather than 22, as in the three-state Porter 5.

### Feedforward neural networks

We defined our baselines implementing window-based FFNN of up to 7 hidden-layers. The symmetric input-window allows segments of AA composed by an odd number of AA, centered on the current n-th position. More in detail, the input at n-th time step is defined as $I(n)={v}_{n-l},{v}_{n-l+1},{v}_{n-l+2},\,\mathrm{..}\,{v}_{n+l}$ where *ν*_*n*_ is the n-th encoded input and *l* is the number of right- and left-adjacent AA considered as additional contextual information, i.e. the input-window contains *l* * 2 + 1AA at any position.

We trained one-hidden-layer FFNN increasing the number of hidden units – to verify whether we had sufficient data to approximate the mapping function (from AA to SS)^58^ – and then trained deeper solutions – i.e. increasing the number of hidden layers. To reduce the computational costs of the hyperparameter search, we adopted an incremental training technique. More in detail, we continued the trainings until completion, then substituted the top layer, i.e. the softmax layer, with untrained hidden-layer + softmax layers and trained these alone, leaving all the weights upstream of them untouched. Finally, we briefly refined the whole FFNN, training every hidden-layer – i.e. end to end, and iterated the process.

### Cascaded bidirectional recurrent and convolutional neural networks

**Figure 1:**
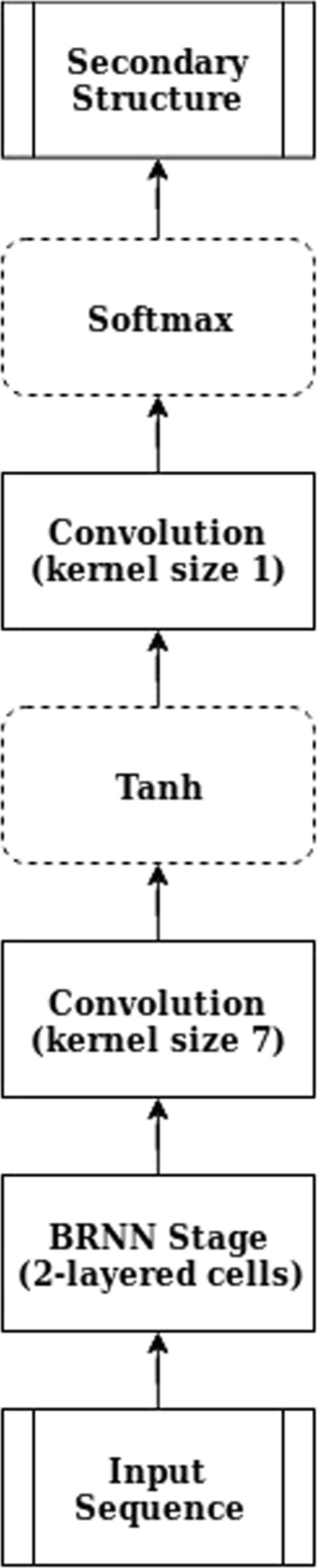
Diagram of the BRCNN. The input sequence is processed by three stages, i.e. one BRNN and two CNN stages, in order to predict the SS. The final architecture of Porter 5 - the CBRCNN - is the (two) cascaded version of the above.

The CBRCNN, assessed in this study and at the core of Porter 5, is an additional refinement of the two-stage bidirectional recurrent neural network (BRNN) initially implemented for the first release of Porter^22^ and successively exploited to predict several more protein structure annotations – e.g. relative solvent accessibility, torsion angles and contact density^59–61^. The CBRCNN preserves two cascaded stages, both containing a BRNN layer with two-layered recurrent cells, and introduces convolutional layers downstream of the BRNN to process windows of both forward and backward chain memories. Differently from the window-based FFNN, the CBRCNN fetches one input/AA at time but then elaborates the entire protein into two Markovian chains (of the BRNN), before processing windows of them through the convolutional layers (see Fig. 1).

In particular, a BRNN (with independent weights and one hidden layer) is followed by a 1D convolutional layer with kernel size greater than one – i.e. able to look at different time steps of the two preceding chain states –, then by a further convolutional layer of kernel size one with softmax outputs. Equivalently, the two convolutional stages can be thought of as a single map implemented by a two-layered network. The output of this overall network is then fed to a similar network for the second stage. The main differences between the first and the second stage are the network size and input: all the layer sizes in the second stage are half the size of those in the first stage, and the output of the first stage network is averaged in different segments to feed the second stage. In other words, the second stage CBRCNN learns to associate every target with a given number of segments, which are built averaging the output of the first stage CBRCNN.

We fixed the number of time steps seen by the first convolutional layer (i.e. the kernel size) to 7 – i.e. 3 adjacent steps per side plus the one at a given position –, and the number of segments of the second stage and their size to 15 and 21, respectively. Therefore, the second stage processes 15 windows, each containing the average of the first stage predictions over 21 time steps, for a total of 315 adjacent steps processed per prediction.

The number of hyperparameters to set in a BRNN, and the more sophisticated internal dynamics, makes this architecture a more complex neural network to train and tune with respect to a FFNN. Step by step, the memory size for the recurrent networks (NF/B), the hidden layer sizes for the recurrent networks (NHF/B) and for the layer preceding the softmax (NHY), in addition to the number of time steps seen by the convolutional layer (CoF/B) and the number and size of the segments feeding the second stage (Cseg and Cwin), have to be determined. The values for these hyperparameters in the models used within Porter 5 are reported in Table 10.

**Table 10:** The hyperparameters of the models employed for Porter 5.

Input	3-state				8-state			
	PSI-BLAST or HHblits			Concatenated	PSI-BLAST or HHblits			Concatenated
NF/B	25	30	30	25	30	35	36	30
NHF/B	40	40	45	40	45	55	60	45
NHY	50	50	55	50	50	45	48	50
CoF/B	3	3	3	3	3	3	3	3
Cseg	10	10	10	10	10	10	10	10
Cwin	7	7	7	7	7	7	7	7

A total of 7 models are ensembled in both 3- and 8-state component. The PSI-BLAST or HHblits only models share the same hyperparameters.

### Ensembling

In all cases we ensembled models by simply taking the average of their class (softmax) outputs. In preliminary tests we briefly assessed more complex strategies, e.g. Bayesian Model Combination^62,63^ in which model-specific weights are learned, but did not find evidence that they performed significantly better than the simple average.

### Measuring performances

The two most commonly used measures to assess SS predictors, accuracy and SOV, have been employed in this study. Accuracy is simply the fraction of AA whose predicted SS class is the same as the observed class, as determined by DSSP^38^. For the 3-class problem (helix, sheet, and coil) we call this Q3 accuracy. For the 8-class problem (*α*-helix, 3_10_-helix, *π*-helix, *β*-sheet, extended strand, hydrogen bonded turn, bend, and other) we call this Q8 accuracy. The 3 classes in the 3-class problem are obtained by merging DSSP-assigned *α*-helix, 3_10_-helix and *π*-helix into class helix, *β*-sheet and extended strand into sheet, and the rest into coil.

We also measured the Segment Overlap (SOV) between the predicted SS and the true one. This latter measure is meant to evaluate the prediction from a more biological viewpoint considering segments rather than single AA as the relevant prediction units. We measured both SOV’99^40^ and SOV_refine^41^.

### Optimization

We implemented momentum^64^ and a dynamic adaptive learning rate to optimize the training process, along with standard stochastic gradient descent (SGD)^65^. We set momentum to 0.9 and divided by two the learning rate any time that the cross entropy error on training set had not decreased for 100 epochs. The training set is shuffled at the end of each epoch, while the size of a mini-batch is set to ~10 proteins, that is, the network weights are updated during training after estimating the gradient on ~10 proteins at a time.

## Data Availability

Porter 5 is available as a web server and light standalone program at http://distilldeep.ucd.ie/porter/ alongside with all the datasets and alignments.
